# A Carbon Fixation Enhanced *Chlamydomonas reinhardtii* Strain for Achieving the Double-Win Between Growth and Biofuel Production Under Non-stressed Conditions

**DOI:** 10.3389/fbioe.2020.603513

**Published:** 2021-01-12

**Authors:** Zhen Zhu, Huijiao Cao, Xu Li, Junfeng Rong, Xupeng Cao, Jing Tian

**Affiliations:** ^1^School of Bioengineering, Dalian Polytechnic University, Dalian, China; ^2^State Key Laboratory of Catalysis, Dalian Institute of Chemical Physics, Chinese Academy of Sciences, Dalian, China; ^3^Division of Solar Energy, Dalian National Laboratory of Clean Energy, Dalian Institute of Chemical Physics, Chinese Academy of Sciences, Dalian, China; ^4^Dalian Key Laboratory of Energy Biotechnology, Dalian Institute of Chemical Physics, Chinese Academy of Sciences, Dalian, China; ^5^Research Centre of Renewable Energy, Research Institute of Petroleum Processing, Sinopec, Beijing, China

**Keywords:** biofuel, *Chlamydomonas reinhardtii*, nitrogen depletion, carbon fixation, photosynthesis, algal station, fatty acids

## Abstract

The stressed cultivations are widely used in microalgae R&D for the biofuel production with the repress on growth to a certain degree, which limits the overall productivity. The balance between the growth and energy storage compounds accumulation is a target needing the combination of both strain selection or construction and culture optimization. Here, an engineered strain of *Chlamydomonas reinhardtii*, in which the chloroplast type glyceraldehyde-3-phosphate dehydrogenase (cGAPDH) was overexpressed and named as P3-GAPDH, was cultured on the Algal Station platform. Compared with wild type (WT), *C. reinhardtii* CC137c, in Tris-acetate-phosphate (TAP) medium, the highest density of WT and P3-GAPDH were 1.23 ± 0.13 and 1.74 ± 0.09 g L^–1^ within 96 h, and the maximum biomass productivity was 24.30 ± 1.65 and 28.54 ± 1.43 mg L^–1^ h^–1^, respectively. In terms of the energy storage compounds, both carbohydrate and fatty acids content doubled in P3-GAPDH, from 0.13 ± 0.02 to 0.26 ± 0.04 g L^–1^ for carbohydrate and from 0.08 ± 0.01 to 0.16 ± 0.01 g L^–1^ for fatty acids, among which poly unsaturated fatty acids increased by 65.8%. Together with the continuous monitor of the chlorophyll fluorescence dynamics parameters *F*_v_/*F*_m_ and *F*_v_’/*F*_m_’ and pH of culture, enhanced Calvin cycle by overexpressed cGAPDH promoted the carbon conversion and subsequent energy storage compounds accumulation. *C. reinhardtii* P3-GAPDH strain showed the potential as a good chassis with high carbon conversion ability.

## Introduction

The green algae *Chlamydomonas reinhardtii* is a model organism that has been systematically studied for more than 50 years (Fields et al., 2018). It has the most abundant physiological and biological information among microalgae with well-annotated genome information, and its genome of chloroplasts and mitochondria has also been sequenced (Harris, 2001). It’s a good candidate for biohydrogen and biofuels development under certain stressed conditions, such as sulfur or nitrogen depletion condition (Faraloni et al., 2011; Gargouri et al., 2017). As a photosynthetic unicellular model organism, it has the advantage of both plant and microbial expression systems, providing the most abundant gene engineering operation possibilities in synthetic biology (Merchant et al., 2007).

As a single cell photosynthetic organism, *C. reinhardtii* provides a platform for the production of a wide range of complex proteins, pigments, and energy storage compounds, and is increasingly recognized as a cheap, scalable, and safe “cell factory” of high-value products as well as bioenergy (Carrera Pacheco et al., 2018). Till now, on the bioenergy and carbon sequestration aspect, *C. reinhardtii* has been investigated on the mechanism and application in hydrogen production (Degrenne et al., 2010; Scoma et al., 2012; Chen et al., 2014), biodiesel production (Fan and Zheng, 2017; Yang et al., 2018) and wastewater treatment (Faraloni et al., 2011), etc.

Like other microalgae, nitrogen depletion stress is a popular method to improve the carbohydrate and triacylglycerols (TAG) content of *C. reinhardtii*. However, the overall biomass productivity was limited due to the inhibition of the growth under stress conditions. Aiming the carbon sequestration and biofuel production, it is necessary to formulate cultivate strategies to balance the energy storage compounds and cell growth to achieve sustainable production with the maximum time-space yield (Moon et al., 2013). By selecting the appropriate cultivation mode and medium, the synchronized proteins, starch, and oils production with cell growth were also achieved in microalgae (Duong et al., 2015a,b; Wang et al., 2015; Zhang et al., 2019; Kim et al., 2020). Besides optimization of the cultivation, screening, or constructing new strains, which are more suitable for guided production under the controlled conditions is a fundamental solution. Because some intrinsic metabolism consumption for stress response is not necessary furthermore in the controlled cultivation and the recycle of this part of carbon and energy could provide the biomass accumulation 20–50% more, such as shift the carbon of photorespiration to carbon fixation in tobacco resulting a 40% increase in biomass productivity (South, 2019). In our previous work, by overexpression of chloroplast glyceraldehyde-3-phosphate dehydrogenase (cGAPDH) in *C. reinhardtii*, the continuous energy storage compounds accumulation was achieved in the flask cultivation (Zhu et al., 2019). cGAPDH is the key enzyme in the Calvin cycle and the joint of carbon fixation and carbon metabolism in the chloroplast. However, the mechanism underlying is unknown. Especially, the previous evaluations were carried out in the flasks with seldom shakes, in which the CO_2_ supply was mostly based on the diffusion from the air and the performance under CO_2_ sufficient conditions was unknown.

Here, the above *C. reinhardtii* strain, P3-GAPDH, was investigated by continuously monitoring its chlorophyll fluorescence dynamics, together with the fatty acids profiling changes to make a deeper understanding of physiological and biochemical aspects. Briefly, the *C. reinhardtii* CC137c (WT) and the dominant P3-GAPDH were parallelly evaluated by self-developed the Algal Station (AS) platform (Cao et al., 2019) in 1.5 L bubbling flat-plate bioreactors under the mixotrophic cultivation modes in TAP medium. AS platform was developed by the Dalian Institute of Chemical Physics, Chinese Academy of Sciences, and it’s the first commercial system to use the maximum quantum yield of photosynthesis II, *F_v_/F_m_*, as an online control parameter for microalgae cultivation together with light intensity, optical density (OD), pH, etc., and meets the requirement from lab to industry by South China University of Technology, Clean Energy Development Center of ENN Group and Microalgae Biotechnology Center of SDIC. The online monitoring of chlorophyll fluorescence dynamics, pH and OD, together with offline measurement of fatty acids profile were carried out to make a detailed evaluation.

## Materials and Methods

### Microalgal Strains and Medium

Microalgal strain *C. reinhardtii* CC137c (wild type, WT) was provided by the Chlamydomonas Resource Center at Duke University. The chloroplast Glyceraldehyde-3-phosphate dehydrogenase (cGAPDH) overexpressed strain *C. reinhardtii* P3-GAPDH was obtained in our previous work (Zhu et al., 2019). Both strains were cultured in TAP media (Kosourov et al., 2007). The seed cells were first cultured in 100 mL conical flasks with 50 mL media. After 3 days, the cells were transferred into 3 L conical flasks with 1 L medium, respectively, under a 14/10 h light/dark cycle with the illumination of the white fluorescent lights at 50 μmol m^–2^ s^–1^ as seeds for bioreactor culture. The temperature was maintained at 25 ± 1°C.

### Cultivation and Sampling

Both WT and P3-GAPDH were cultured for 96 h in a 1.5 L flat-plate poly (polymethyl methacrylate) (PMMA) photobioreactor (15.2 × 3.5 × 30 cm in length, width, and height) on Algal Station (AS) system, which was aired with 2% CO_2_ compressed air as described previously (Cao et al., 2019). The aeration rate was 0.4 vvm and supplied throughout the culture. The light intensity on the front surface of the flat-plate was 300 μmol m^–2^ s^–1^ under the 14/10 h light/dark cycle, in which the illumination started at 8:00 am and ended at 10:00 pm. The culture temperature was 25 ± 1°C. The inoculation was at 3:00 pm at Day 0 and the samples were taken at 9:00 am and 9:00 pm every day for offline verification of OD and *F_v_/F_m_*, as well as for *F*_v_’*/F_m_*’, carbohydrate and fatty acid profile determination after the centrifugation. Three batches experiments were carried out. The online monitoring of OD and *F*_v_/*F*_m_ were taken with a 20 min interval.

To make it easily to understand the effect of light cycle during the culture, the time scale used here were based on light/dark cycle, which caused the inoculation started at the point of 7 h relatively, and similarly hereinafter without specification.

### Optical Density and Dry Weight

To ensure the OD detection by non-diluted measurement with AS, the offline verification was carried out by a UV/VIS spectrophotometer (Jasco V-650, JASCO Corporation, Japan) at 750 nm. For the detection by Jasco V-650, the samples were diluted to keep a reliable OD_750_ under 1.0.

The specific growth rate (μ) was calculated in terms of the Equation (1):

(1) $$\mu =\left(l\,n\,O\,D_{2}-l\,n\,O\,D_{1}\right)/12$$

The OD_1_ and OD_2_ are the absorbances by the AS with a 12 h’s interval at the same time as the offline sampling, respectively.

The dry weight was measured by filtering a certain volume (5–10 mL) of cultures by pre-dried and pre-weighed Whatman GF/C filters dried at 60°C for 24 h as previous report (Meng et al., 2015). The dry weight was calculated in terms of the Equation (2) with three replicates:

(2) $$D\,W=\left(w_{2}-w_{1}\right)/v$$

where w_1_ is the weight of pre-dried GF/C filter in g, w_2_ is the weight of filter with dried algal culture in g, and v is the initial volume of the sample in L.

The productivity was calculated by the equation:

(3) $$P=1000*\left(D\,W_{2}-D\,W_{1}\right)/12$$

where DW_1_ and DW_2_ are the corresponding dry weight in g L^–1^ with a 12 h’s interval, respectively, and P is the productivity in mg L^–1^ h^–1^.

### Chlorophyll Fluorescence Dynamics Parameters Detection

The online monitoring of *F_v_/F_m_* by AS with 20 min interval showed a continuous change pattern and it was also verified by a chlorophyll fluorometer (Water-PAM Heinz Walz GmbH, Effeltrich, Germany) following the procedure in previous reports (Yao et al., 2012, 2013) during for the offline sampling. The effective photochemical quantum yield, *F*_v_’*/F_m_*’ was simultaneously detected by Water-PAM together with *F_v_/F_m_*.

### Carbohydrate Analysis

Carbohydrate content was determined by the colorimetric method of anthrone-sulfuric acid as described previously (Zhu et al., 2019). In brief, about 5 mg of dried biomass was weighed and ultrasonic crushing followed by treatment in boiling water for 10 min with pre-prepared anthrone-sulfuric acid (74%). The quantifications were carried out on UV/VIS spectrophotometer mentioned before against the standard curve of “glucose concentration—absorbance,” which was shown in Supplementary Material.

### Fatty Acid Analysis

The fatty acid profile based on fatty acid methyl esters (FAMEs) were detected by GC after a quick transesterification with methanol following previous report (Yang et al., 2017). In brief, about 5 mg dried biomass was transesterification in 5 mL methanol with 2% H_2_SO_4_ at 70°C for 1 h. Then 2 mL hexane together with 0.75 mL deionized water were used to extract FAMEs and the obtained hexane mixture was further dewatered by about 0.5 g anhydrous Na_2_SO_4_. Then the mixture was analyzed on an Agilent 7890 GC with a DB-23 capillary column (30 mm × 0.32 mm × 0.25 μm, Agilent Technology) and a flame ionization detector (FID). The injector temperature was 270°C with a split ratio of 50:1. The column was heated at 130°C for 1 min, then increased to 170°C at a rate of 10°C min^–1^, and then increased at a rate of 2.8°C min^–1^ to 215°C where it was maintained for 1 min. After the determination of FA profiling, the group of saturated/monounsaturated/polyunsaturated FAs were summing up and the percentage of them in the total FAs were calculated.

### Statistical Analysis

All data were presented as the averages ± *SD* for three biological replicates from three batches. The One-way ANOVA analyses were performed in Excel (version 2016, Microsoft) to make the significance analysis for the fatty acid content. The *P*-value was obtained through data analysis in Excel. The level of statistical significance was set to a significant difference (*p* < 0.05) and an extremely significant difference (*p* < 0.01).

## Results

### Growth and Biomass Accumulation

**FIGURE 1:**
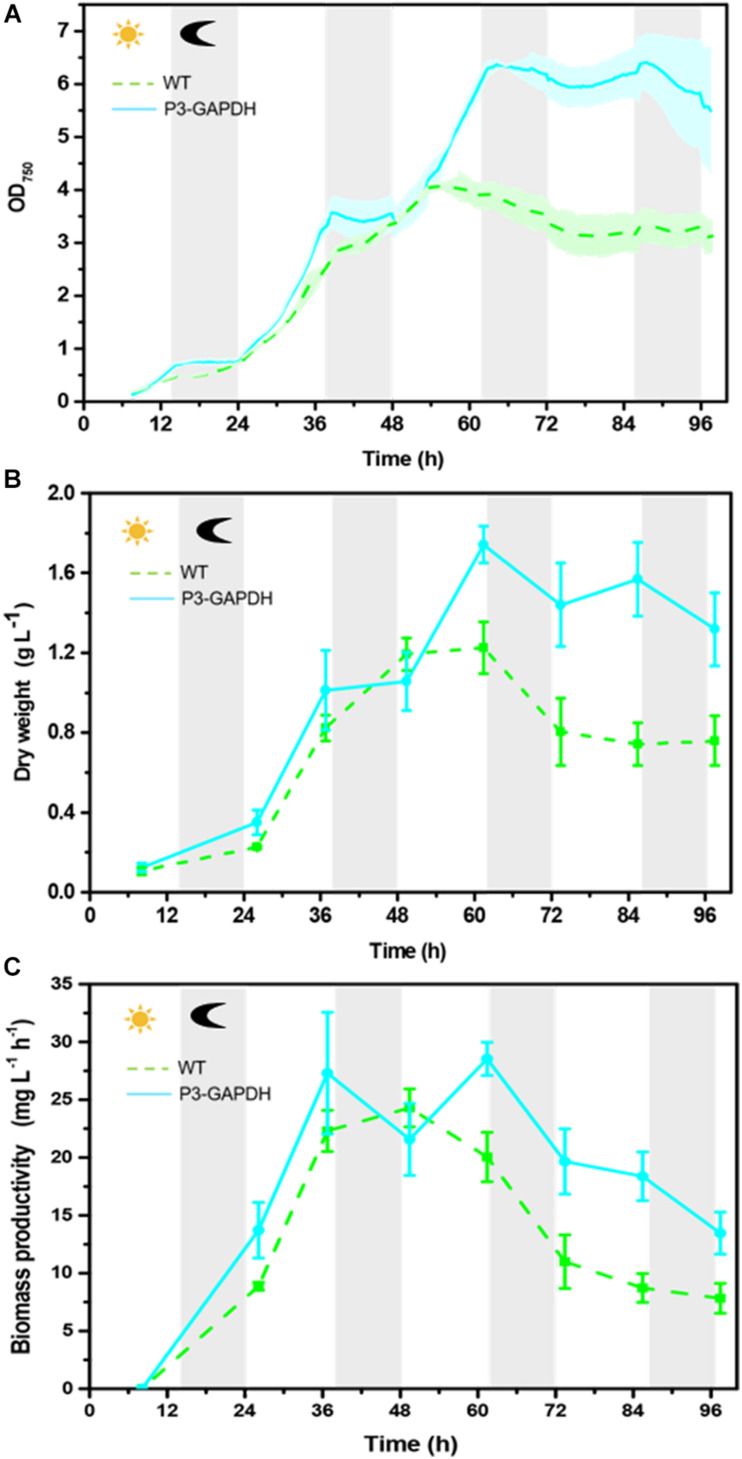
The growth difference between two strains. **(A)** Growth curve by Algal Station, **(B)** Dry weight curve, **(C)** Biomass productivity curve of WT and P3-GAPDH during light-dark cycles. The productivity is the biomass per unit time. Line represents P3-GAPDH, dot line represents WT, and shaded areas (in **A**) or error bars (in **B**,**C**) of each line indicate the standard error of the line values. The gray background marked with a dark moon represents the dark time, while the white background marked with a sun represents illumination during the cultivation. The time scale used here were based on light/dark cycle, which caused the inoculation started at the point of 7 h in the time scale.

The growth in P3-GAPDH was monitored and compared with WT on the AS platform for 4 days (Figure 1A and Supplementary Table S1). There was no significant difference in the growth during the first 2 days, while P3-GAPDH showed a clear step like day-night change pattern. P3-GAPDH’s OD was almost flat in the dark, whereas increased rapidly with illumination. The maximum OD of WT reached 4.08 at 50 h and there were no difference in OD between WT and P3-GAPDH before it. The WT turned into the steady phase then. However, P3-GAPDH kept growing to 60 h and its maximum OD reached 6.37, which increased more than 50% than that of WT. The maximum specific growth rates (μ_max_) reached at 30 h for both P3-GAPDH and WT, were 0.13 and 0.09 h^–1^, respectively. By the way, the data for Figure 1A were collected directly from AS with a 20 min intervals, and the variation between batches were indicated by shadows for each strain.

The dry weights showed a similar trend to OD and were shown in Figures 1B,C. The maximum DW of WT and P3-GAPDH were 1.23 ± 0.13 and 1.74 ± 0.09 g L^–1^, respectively, with biomass productivity of 24.30 ± 1.65 and 28.54 ± 1.43 mg L^–1^ h^–1^. Compared with previous reports of the mixotrophic cultivation of *C. reinhardtii* in Table 1, P3-GAPDH had the highest productivity in biomass under similar conditions.

**TABLE 1:** Comparison of *C. reinhardtii* mixotrophy cultivation among this study and other reports.

***C. reinhardtii* strains**	**Bioreactor**	**Light intensity (μ molm^–2^ s^–1^)**	**Photoperiod (h)**	**Medium**	**Dry biomass per culture (g L^–1^)**	**Biomass productivity (mg L^–1^ h^–1^)**	**References**
UTEX-90	100 L flat-vertical photobioreactor	n.r	Outdoor cultivation	TAP+2%CO_2_	1.45	20.13	Kim et al., 2006
137C	1.5 L torus PBR/5-L cylindrical PBR	500	n.r	TAP	1.1/0.9	4.6	Degrenne et al., 2010
CC124	0.5 L glass columns	70 (both sides)	24	TAP+3%CO_2_	1.7	26	Faraloni et al., 2011
CC124	Outdoor 50 L horizontal tubular photobioreactor	500 (both sides)	Outdoor cultivation	TAP+3%CO_2_	1.6	9.5	Scoma et al., 2012
CC124	250 mL flasks	180–200	n.r	TAP+10 g/L acetate	2.15	18	Moon et al., 2013
CC124/CC125 mutant	250 mL flasks	180–200	6/18	TAP	1.1/2.1	9.16	Carrera Pacheco et al., 2018
CC137c	1.5 L flat-plate poly(polymethyl merthacrylate) PBR	300	14/10	TAP+2%CO_2_	1.23	24.3	This study
P3-GAPDH	1.5 L flat-plate poly(polymethyl merthacrylate) PBR	300	14/10	TAP+2%CO_2_	1.74	28.54	This study

*n. r not reported.*

### *F_v_/F_m_* and *F*_v_’*/F_m_*’ Patterns

**FIGURE 2:**
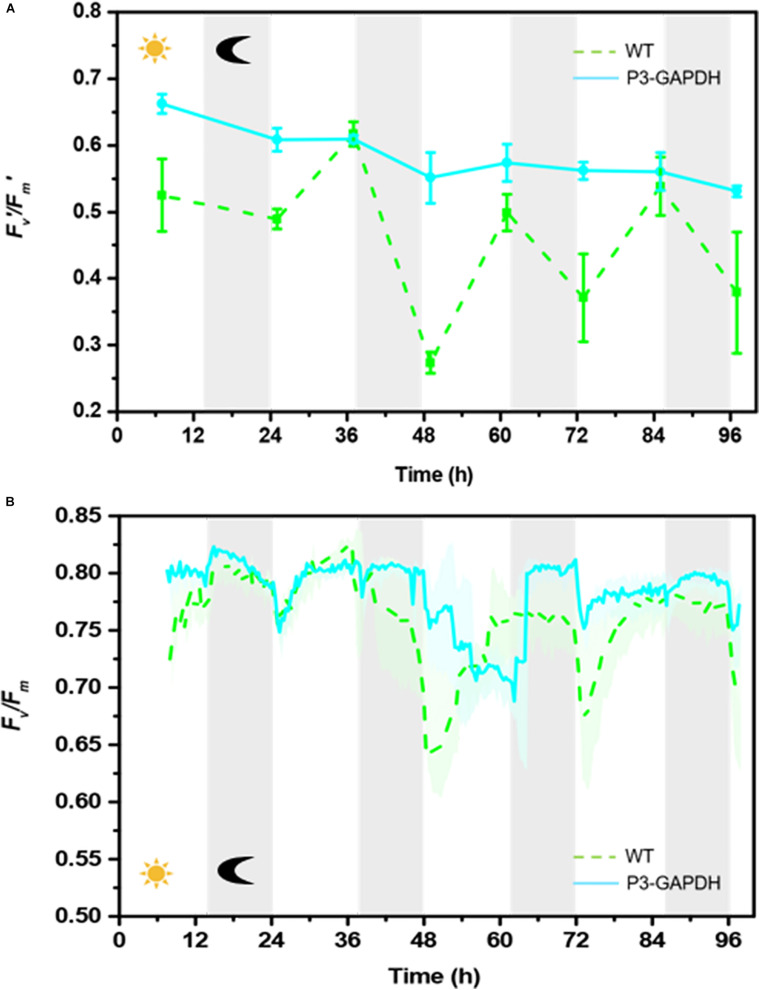
The *F_v_’/F_m_’* and *F_v_/F_m_* change patterns difference. **(A)** *F_v_’/F_m_’* of WT and P3-GAPDH by water-PAM, **(B)** *F_v_/F_m_* of WT and P3-GAPDH by Algal Station. Line represents P3-GAPDH, dot line represents WT, and shaded areas (in **B**) or error bars (in **A**) of each line indicate the standard error of the values. The gray background marked with a dark moon represents the dark time, while the white background marked with a sun represents illumination during the cultivation.

The chlorophyll fluorescence dynamics parameters, the maximum quantum of PSII, *F_v_/F_m_*, and the effective photochemical quantum yield of PSII, *F_ v_’/F_m_’*, changes were shown in Figure 2 (Supplementary Table S2). The *F_v_’/F_m_’* of P3-GAPDH were higher and more stable than that of WT in almost all the whole cultivation, which indicated the higher photochemical conversion in P3-GAPDH. The *F_v_’/F_m_’* of WT showed a more fluctuation pattern with the light cycle, increasing with the illumination and decreasing in the dark after day 1, and lowed to 0.27 after the second dark period. On the contrary, the *F_v_’/F_m_’* kept above 0.5 throughout the P3-GAPDH’s cultivation and there was less correlation to the light cycle as WT.

The overall pattern of *F_v_/F_m_* in both strains showed obvious light dependent pattern with the help of continuously monitoring by AS in Figure 2B. Without light, the *F_v_/F_m_* kept stable in the dark. While the illumination started, it dropped quickly followed by a relatively slow recovery in both two strains except in the third light period. As the OD curves in Figure 1A, the *F_v_/F_m_* data were collected by AS and the variations were shown as shadows.

From both Figures 2A,B, the obviously fluctuation happened in the third light period, which may link to the change of mixtrophic cultivation to autotrophic cultivation and will be discussed later.

### Carbohydrate Content Changes

**FIGURE 3:**
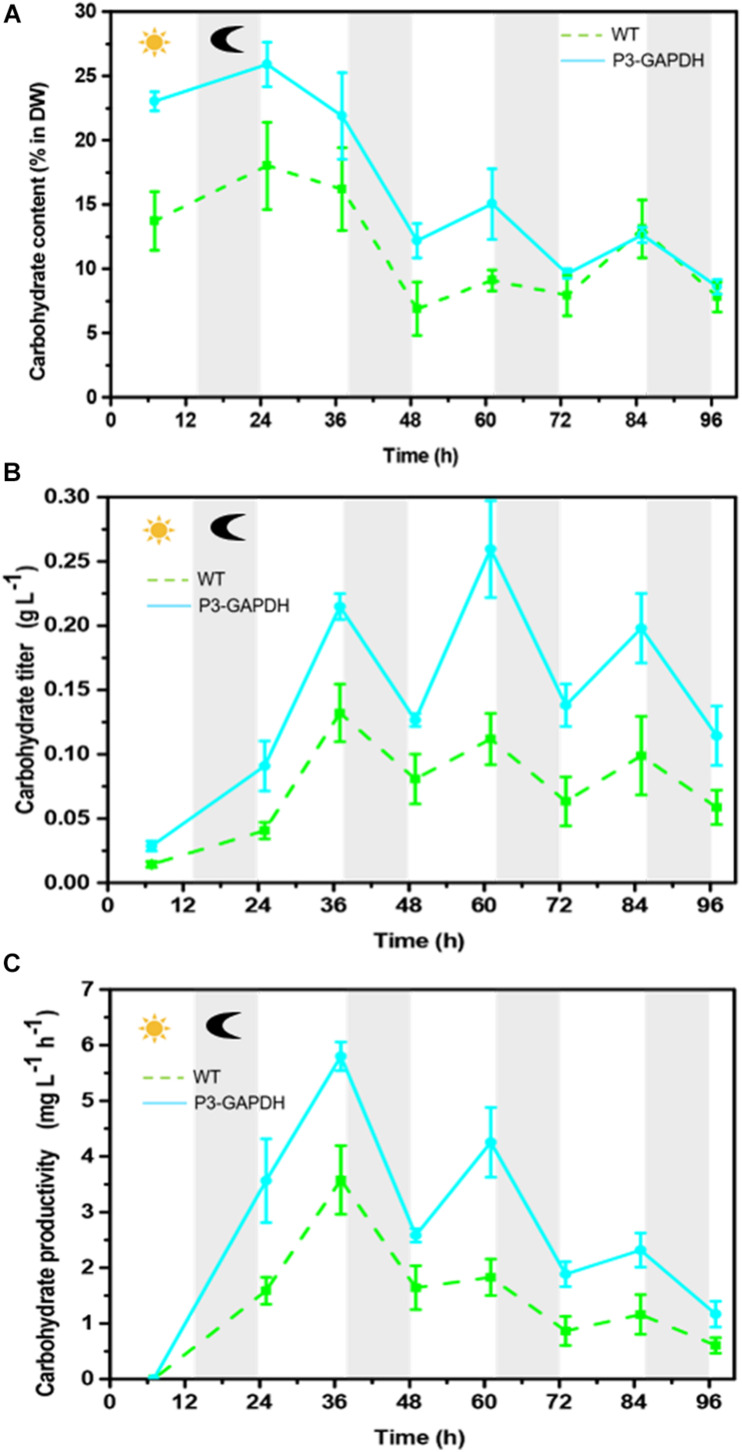
The difference in carbohydrate content and production. **(A)** Carbohydrate content, **(B)** carbohydrate yield, **(C)** carbohydrate productivity of WT and P3-GAPDH. Carbohydrate productivity is the yield per unit time. Line represents P3-GAPDH, dot line represents WT, and error bars indicate the standard error of the values. The gray background marked with a dark moon represents the dark time, while the white background marked with a sun represents illumination during the cultivation.

The carbohydrate content and production of P3-GAPDH kept higher than those of WT, with a large fluctuation ranges with the illumination cycle (Figure 3). On average, the carbohydrate content in WT was 16.2% of dry weight, with the highest titer of 0.13 ± 0.02 g L^–1^ and a maximum productivity of 3.58 mg L^–1^h^–1^. Comparing to WT, carbohydrate accounted for 25.9% of the dry weight in P3-GAPDH at time point 25 h, and the maximum titer was 0.26 ± 0.04 g L^–1^, with a maximum productivity of 5.80 mg L^–1^h^–1^. The high carbohydrate titer in P3-GAPDH in the bioreactor cultivation was consistent with the previous report in the flask (Zhu et al., 2019). By the overexpression of cGAPDH, the carbohydrate productivity nearly doubled under the light within the third and the forth cycle.

### Fatty Acid Content and Profiling Changes

**FIGURE 4:**
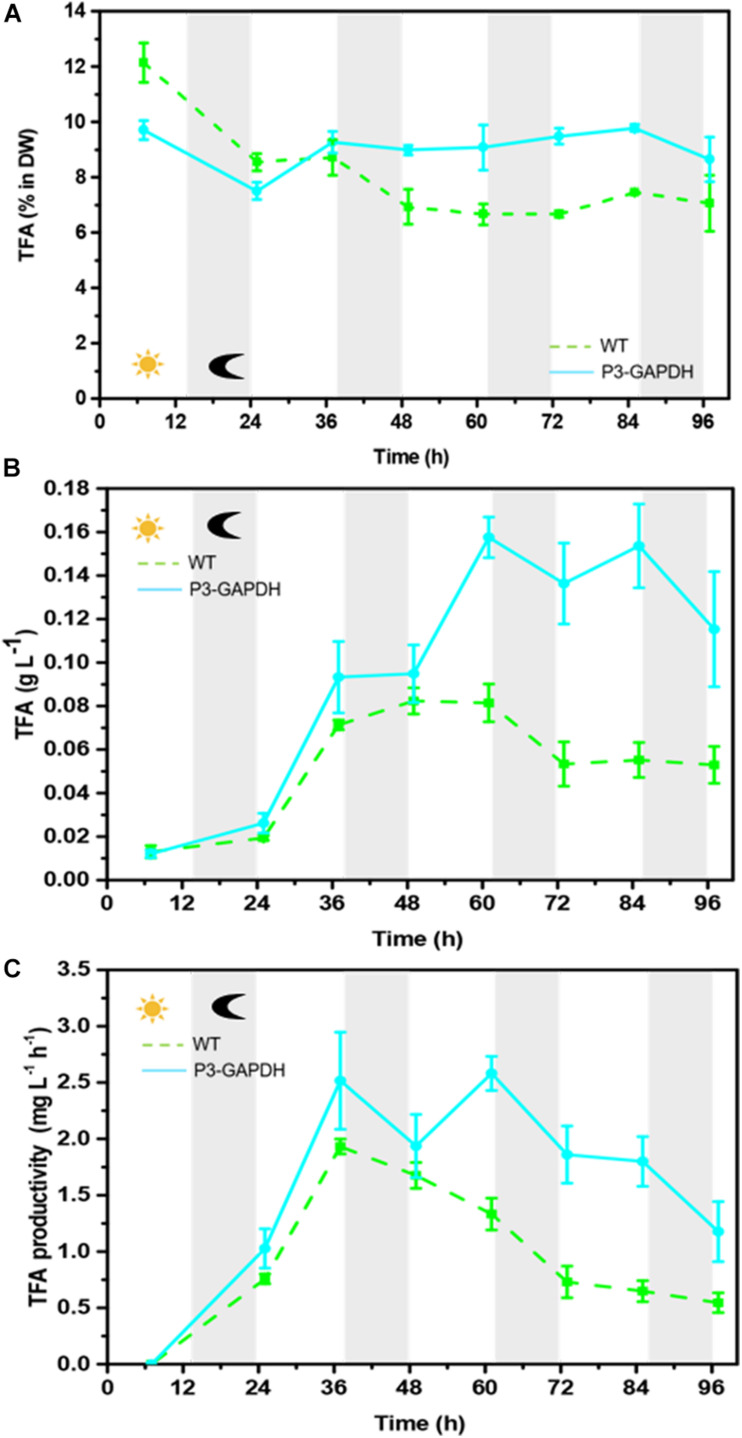
The difference in TFA content and production. **(A)** TFA content, **(B)** TFA yield, **(C)** TFA productivity of WT and P3-GAPDH. TFA productivity is the yield per unit time. Line represents P3-GAPDH, dot line represents WT, and error bars indicate the standard error of the values. The gray background marked with a dark moon represents the dark time, while the white background marked with a sun represents illumination during the cultivation.

By the sum of each FA’s content detected by GC (Table 2), both the fatty acid (FA) content and production were compared between WT and P3-GAPDH and shown in Figure 4. Although the total FA (TFA) content of WT was higher than P3-GAPDH at the inoculation, the TFA content in WT decreased from 12.1 to nearby 7.0% in the steady phase. The TFA content maintained a relatively stable level in P3-GAPDH nearby 10.0%, except a drop to 7.3% at time point 25 h (Figure 4A). However, due to more biomass produced in P3-GAPDH, the P3-GAPDH’s TFA yield nearly doubled while compared to that in WT, 0.16 ± 0.01 and 0.08 ± 0.01 g L^–1^, and with the maximum productivity of 2.58 ± 0.15 and 1.93 ± 0.07 mg L^–1^ h^–1^, respectively (Figures 4B,C).

**TABLE 2:** Fatty acid composition and the proportion of SFA, MUFA, PUFA in TFA of WT and P3-GAPDH grown in TAP medium after 96 h.

	**7 h**		**24 h**		**36 h**		**48 h**		**60 h**		**72 h**		**84 h**		**96 h**	
	**WT**	**P3-GAPDH**	**WT**	**P3-GAPDH**	**WT**	**P3-GAPDH**	**WT**	**P3-GAPDH**	**WT**	**P3-GAPDH**	**WT**	**P3-GAPDH**	**WT**	**P3-GAPDH**	**WT**	**P3-GAPDH**
C16:0	2.33 ± 0.10	2.00 ± 0.05	1.90 ± 0.05	1.82 ± 0.03	1.73 ± 0.16	2.00 ± 0.05	1.64 ± 0.18	2.11 ± 0.11	1.57 ± 0.03	2.22 ± 0.02	1.59 ± 0.04	2.29 ± 0.15	1.75 ± 0.08	2.19 ± 0.09	1.69 ± 0.11	2.03 ± 0.10
C16:1n9	0.66 ± 0.04	0.49 ± 0.02	0.27 ± 0.04	0.21 ± 0.01	0.33 ± 0.00	0.33 ± 0.02	0.25 ± 0.02	0.34 ± 0.04	0.33 ± 0.03	0.37 ± 0.07	0.44 ± 0.07	0.42 ± 0.01	0.45 ± 0.04	0.49 ± 0.03	0.46 ± 0.09	0.47 ± 0.01
C16:1n7	0.23 ± 0.02	0.21 ± 0.01	0.20 ± 0.02	0.17 ± 0.01	0.44 ± 0.10	0.18 ± 0.01	0.81 ± 0.10	0.28 ± 0.07	0.67 ± 0.04	0.32 ± 0.14	0.48 ± 0.02	0.20 ± 0.02	0.38 ± 0.02	0.21 ± 0.01	0.36 ± 0.04	0.24 ± 0.04
C16:2n6	0.35 ± 0.01	0.21 ± 0.00	0.08 ± 0.01	0.04 ± 0.00	0.09 ± 0.00	0.04 ± 0.01	0.03 ± 0.00	0.05 ± 0.01	0.07 ± 0.00	0.12 ± 0.03	0.08 ± 0.02	0.15 ± 0.03	0.14 ± 0.02	0.18 ± 0.03	0.11 ± 0.00	0.12 ± 0.01
C16:3n6	0.30 ± 0.04	0.17 ± 0.01	0.14 ± 0.01	0.08 ± 0.00	0.11 ± 0.01	0.08 ± 0.01	0.06 ± 0.01	0.07 ± 0.01	0.07 ± 0.01	0.07 ± 0.02	0.09 ± 0.02	0.09 ± 0.01	0.13 ± 0.02	0.13 ± 0.01	0.11 ± 0.02	0.10 ± 0.01
C16:3n3	0.20 ± 0.02	0.14 ± 0.00	0.14 ± 0.02	0.11 ± 0.01	0.16 ± 0.05	0.20 ± 0.03	0.06 ± 0.02	0.13 ± 0.01	0.07 ± 0.02	0.16 ± 0.04	0.06 ± 0.01	0.15 ± 0.01	0.10 ± 0.01	0.18 ± 0.03	0.09 ± 0.03	0.16 ± 0.03
C16:4n3	1.61 ± 0.20	1.35 ± 0.04	0.95 ± 0.06	0.86 ± 0.03	1.10 ± 0.11	1.27 ± 0.07	0.65 ± 0.12	1.19 ± 0.08	0.68 ± 0.14	1.14 ± 0.24	0.74 ± 0.05	1.26 ± 0.02	0.89 ± 0.08	1.39 ± 0.06	0.87 ± 0.26	1.19 ± 0.18
C18:0	0.31 ± 0.01	0.33 ± 0.02	0.27 ± 0.02	0.23 ± 0.01	0.24 ± 0.03	0.26 ± 0.00	0.21 ± 0.03	0.27 ± 0.01	0.19 ± 0.04	0.28 ± 0.02	0.20 ± 0.03	0.33 ± 0.05	0.19 ± 0.03	0.27 ± 0.03	0.21 ± 0.04	0.26 ± 0.03
C18:1n9	0.24 ± 0.02	0.20 ± 0.01	0.60 ± 0.02	0.38 ± 0.02	0.58 ± 0.03	0.39 ± 0.10	0.30 ± 0.03	0.31 ± 0.06	0.20 ± 0.06	0.22 ± 0.02	0.13 ± 0.02	0.18 ± 0.02	0.12 ± 0.01	0.11 ± 0.01	0.12 ± 0.02	0.10 ± 0.01
C18:1n7	0.54 ± 0.01	0.33 ± 0.02	0.49 ± 0.03	0.34 ± 0.01	0.49 ± 0.03	0.43 ± 0.00	0.86 ± 0.04	0.51 ± 0.05	0.80 ± 0.04	0.55 ± 0.07	0.65 ± 0.02	0.46 ± 0.05	0.62 ± 0.08	0.55 ± 0.04	0.60 ± 0.10	0.51 ± 0.08
C18:2n6	1.68 ± 0.03	1.13 ± 0.02	0.69 ± 0.02	0.59 ± 0.03	0.69 ± 0.06	0.59 ± 0.10	0.41 ± 0.08	0.52 ± 0.06	0.37 ± 0.03	0.66 ± 0.05	0.43 ± 0.07	0.71 ± 0.07	0.58 ± 0.09	0.62 ± 0.09	0.48 ± 0.06	0.43 ± 0.05
C18:3n6	0.72 ± 0.02	0.63 ± 0.17	0.66 ± 0.03	0.61 ± 0.12	0.57 ± 0.04	0.73 ± 0.23	0.38 ± 0.06	0.63 ± 0.01	0.36 ± 0.04	0.52 ± 0.09	0.39 ± 0.00	0.64 ± 0.05	0.42 ± 0.02	0.67 ± 0.03	0.40 ± 0.08	0.62 ± 0.07
C18:3n3	2.80 ± 0.30	2.35 ± 0.06	1.95 ± 0.11	1.89 ± 0.04	2.03 ± 0.23	2.53 ± 0.10	1.17 ± 0.25	2.30 ± 0.11	1.16 ± 0.24	2.24 ± 0.45	1.23 ± 0.08	2.35 ± 0.01	1.47 ± 0.14	2.48 ± 0.12	1.37 ± 0.41	2.08 ± 0.35
C18:4n3	0.19 ± 0.01	0.16 ± 0.01	0.20 ± 0.02	0.17 ± 0.01	0.16 ± 0.02	0.24 ± 0.01	0.10 ± 0.02	0.25 ± 0.01	0.13 ± 0.03	0.20 ± 0.05	0.16 ± 0.02	0.24 ± 0.02	0.19 ± 0.03	0.31 ± 0.01	0.18 ± 0.06	0.31 ± 0.05

	**7 h**		**24 h**		**36 h**		**48 h**		**60 h**		**72 h**		**84 h**		**96 h**	
	**WT**	**P3-GAPDH**	**WT**	**P3-GAPDH**	**WT**	**P3-GAPDH**	**WT**	**P3-GAPDH**	**WT**	**P3-GAPDH**	**WT**	**P3-GAPDH**	**WT**	**P3-GAPDH**	**WT**	**P3-GAPDH**

SFA	21.8 ± 0.7	24.0 ± 0.4	25.5 ± 0.5	27.3 ± 0.7	22.5 ± 0.5	24.4 ± 0.8	26.6 ± 0.6	26.6 ± 0.8	26.4 ± 0.6	27.7 ± 2.4	26.8 ± 0.3	27.6 ± 1.3	26.1 ± 0.7	25.1 ± 1.3	27.1 ± 1.9	26.6 ± 1.0
MUFA	13.8 ± 0.8	12.6 ± 0.3	18.2 ± 0.2	14.8 ± 0.4	21.3 ± 2.3	14.3 ± 1.0	32.5 ± 5.2	16.0 ± 1.2	30.3 ± 3.2	16.4 ± 3.1	25.6 ± 0.4	13.4 ± 0.9	21.1 ± 1.2	13.9 ± 0.1	22.5 ± 4.2	15.4 ± 1.0
PUFA	64.4 ± 1.3	63.4 ± 0.7	56.3 ± 0.5	57.9 ± 0.9	56.2 ± 1.8	61.3 ± 1.7	40.8 ± 4.6	57.4 ± 2.0	43.3 ± 3.8	55.9 ± 5.2	47.6 ± 0.1	59.0 ± 1.4	52.7 ± 1.9	61.0 ± 1.2	50.4 ± 6.1	58.0 ± 1.9

*Data expressed as a percentage of dry weight (% in DW) and were shown as means−x ± SD (n = 3). Data expressed as a percentage of SFA, MUFA, PUFA in TFA (%) and were shown as means−x ± SD (n = 3).*

**FIGURE 5:**
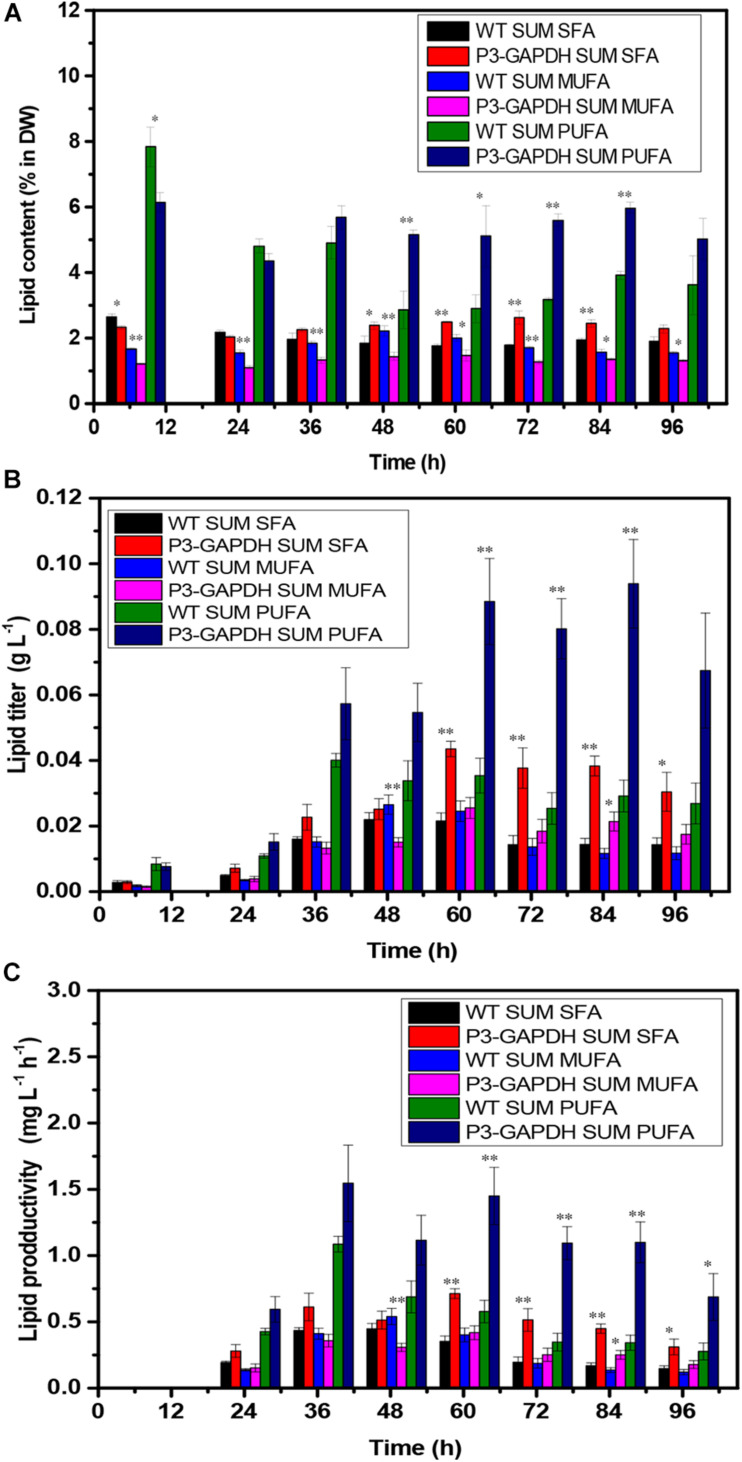
The difference in FAs composition. **(A)** FAs content, **(B)** FAs yield, **(C)** FAs productivity of WT and P3-GAPDH. FAs productivity is the yield per unit time. These fatty acids were classified into three categories: SFA, MUFA, PUFA, and compared with the content for each kind of fatty acid, respectively. For each point, the mean is given, *n* = 3. * and ** denote significant difference (*p* < 0.05) and extremely significant difference (*p* < 0.01) between WT and P3-GAPDH, respectively.

In *C. reinhardtii*, polyunsaturated fatty acids (PUFAs), such as C18:3, construct the majority of the membrane of the chloroplast and thylakoid and contribute to the photosynthesis process (Yang et al., 2017). The contents and titers of saturated fatty acids (SFA), monounsaturated fatty acids (MUFA), and PUFA in WT and P3-GAPDH, which changed significantly different (Figure 5). In P3-GAPDH, more PUFA, such as C16:4, C18:2, C18:3, C18:4, were produced and a stable content were maintained. The maximum titer of SFA and PUFA were 0.02 ± 0.00 and 0.04 ± 0.00 g L^–1^, respectively, in WT, while the maximum titer of SFA and PUFA were 0.04 ± 0.00 and 0.09 ± 0.01 g L^–1^ in P3-GAPDH, which was twofold and 2.25-fold to WT, respectively. The percentage of SFA, MUFA, PUFA in TFA was shown in Table 2. It was clear that PUFA was predominant in TFA. The detailed increment of fatty acids in P3-GAPDH compared with WT was summarized in Table 3. The increased in PUFA production was also consisted with the start of the second growth wave of P3-GAPDH after time point 36 h.

**TABLE 3:** Increment of fatty acids in P3-GAPDH compared with WT.

**Fatty acid**	**61 h**	**73 h**	**85 h**	**97 h**
Total fatty acid (TFA)	93%	155%	178%	118%
Saturated (SFA)	102%	164%	166%	113%
Monounsaturated (MUFA)	4%	35%	84%	49%
Polyunsaturated (PUFA)	150%	216%	222%	151%

### pH Changes of WT and P3-GAPDH During Cultivation

**FIGURE 6:**
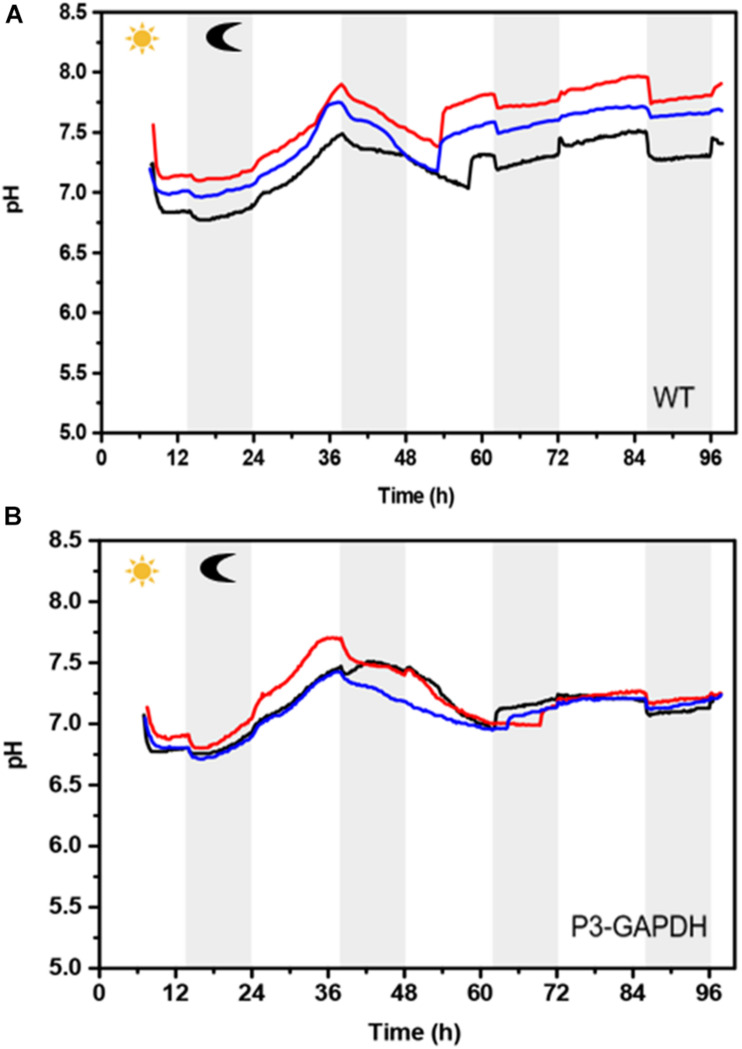
pH changes during the cultivation | pH curve of **(A)** WT and **(B)** P3-GAPDH. Three lines represent three batches.

The pH-values of two microalgae were monitored by AS was significantly different (Figure 6). It’s was notable that between time point 48–62 h, the pH-value of WT showed a bigger fluctuation than that in P3-GAPDH. The change of pH here indicated the depletion of acetate in the TAP and the loss of some buffer ability from it.

## Discussion

GAPDH is the key enzyme in the glycolysis and Calvin cycle. Most studies were focused on the cytoplasm type GAPDH, and the chloroplast GAPDH’s contribution to the carbon metabolism was rarely studied. It has been postulated to play an important role in the supramolecular complexes in the Calvin cycle in the *C. reinhardtii* based on structure biology studies (Emmanuelle et al., 2004; Erales et al., 2009). In *Chlamydomonas*, glycolysis is divided into a chloroplastidic and a cytosolic part with the enzymes GAPDH/phosphoglycerate kinase (PGK) being located in the chloroplast stroma and its activity depends onNADPH mediated complex dissociation under the regulation of the light (Wedel and Soll, 1998). After the identification the significant changes of cGAPDH during the nitrogen starvation in *C. reinhardtii*, the strain with overexpressed cGAPDH in the chloroplast was constructed to evaluate its contribution to carbon fixation previously (Zhu et al., 2019) and results showed a better carbon conversion in the P3-GAPDH strain. However, in the flask cultivation with TAP media, the mixtrophic condition with low CO_2_ supply limited the understanding of the effect of cGAPDH on the carbon fixation. So, a more detailed comparison of P3-GAPDH with WT was carried out in the bioreactor with enriched CO_2_ supply, in which both the mixtrophic and autotrophic cultivation existed. Based on the continuously monitoring of OD and pH, the switch of above two cultivation modes happened nearby the time point 50 h (Figures 1A, 6). To simplify, the culture before 50 h was treated as mixtrophic mode (Mix) and the left as autotrophic mode (Auto).

With the existence of acetate, P3-GAPDH only showed a little help in the growth (OD), However, a more than 50% increase in carbohydrate productivity still achieved (Figure 3C) in the Mix mode and resulted in the highest productivity under the similar cultivation conditions (Tables 1, 4). It’s postulated that overexpressed cGAPDH contributed to more carbon fixation from two aspects: (1) Due to cGAPDH converts the products of carbon fixation in the Calvin cycle, the overexpression of it will also help to reduce the energy or electron accumulation in the electron transfer chain of the light reaction part in the photosynthesis, which was indicated by the relative higher and stable *F_v_’*/*F_m_’* (Figure 2A). The overexpression might break the light regulation of cGAPDH’s activity with extra free enzymes from the GAPDH/PGK complex. It’ll be verified by the proteomics analysis in our further work. (2) The maintenance of the photosynthesis system, and was indicated by the continuously monitoring of *F*_v_/*F*_m_ (Figure 2B). As the major components in the thylakoid membrane, where the photosynthesis carries out, glycolipids [monogalactosyldiacylglycerol (MGDG), digalactosyldiacylglycerol (DGDG), and sulphoquinovosyldiacylglycerol (SQDG)], which are mostly formed with PUFAs (Yang et al., 2018), and P3-GAPDH strain was proved to produced more PUFAs (Figure 5 and Table 2; Zhu et al., 2019).

**TABLE 4:** The comparison among this study and Faraloni et al. (2011).

***C. reinhardtii* strains**	**Temperature**	**Light intensity**	**Dry biomass per culture (g L**^–1^)	**The time of max dry biomass (h)**	**Biomass productivity (mg L^–1^ h^–1^)**	**The time of max biomass productivity (h)**	**Specific growth rates (h**^–1^)
CC124	28°C	70 μmol m^–2^ s^–1^ (supplied on both sides)	1.70	65 (24 h light)	26.0	65	0.08
CC137c	25°C	300 μmol m^–2^ s^–1^ (supplied on one side)	1.23	54 (14/10 h light)	24.3	42	0.09
P3-GAPDH	25°C	300 μmol m^–2^ s^–1^ (supplied on one side)	1.74	54 (14/10 h light)	28.5	54	0.13

Combined both the physiological and biochemical analysis, cGAPDH is postulated to play an important role in the regulation of carbon fixation in the chloroplast. Here, without the repletion of other nutrients, the overexpression of chloroplast GAPDH gene enables the P3-GAPDH to maintain high photosynthetic activity and promote biomass production, i.e. the carbohydrate and lipid content increased by 96.6 and 93.4%, respectively, which were mostly from the Auto mode cultivation. The results indicated that overexpressed cGAPDH increased the carbon fixation capacity of *C. reinhardtii* not only by push the carbon to downstream metabolisms, such as carbohydrates and FAs, but also promote the photochemical conversion in the photosynthesis, liking a self-inspiring cycle: overexpressed cGAPDH producing more C3 metabolite for PUFAs synthesis, while more PUFAs maintaining a more robust photosynthesis and resulting more fixed carbon for cGAPDH to use. It brings the hope to achieve the two-win between growth and energy storage compounds production other than traditional nitrogen depletion stress with the suppress of the growth, i.e., to achieve both higher biomass and higher energy storage compounds production simultaneously.

Here only the physiological and content analysis was carried out. Detailed molecular biology studies, such as—omics analysis, were needed to understand the carbon fixation improvement by overexpressing the cGAPDH to aid the future synthetic biology development by using a microalgae cell factory.

## Conclusion

The cGAPDH overexpressed strain P3-GAPDH was verified in the bioreactor and it higher carbon fixation than previously reported under similar conditions was confirmed. The overexpress cGAPDH not only promoted the carbon conversion from photosynthesis, but also formed a self-inspiring cycle by promoting the PUFAs synthesis, which are important to the maintenance high photosynthesis activity.

## Data Availability Statement

The original contributions presented in the study are included in the article/Supplementary Material, further inquiries can be directed to the corresponding author/s.

## Author Contributions

XC, JT, and JR designed the research. ZZ, HC, and XC wrote the manuscript and analyzed the data. ZZ, HC, and XL performed the research and provided technical support. All authors read and approved the final manuscript.

## Conflict of Interest

The authors declare that the research was conducted in the absence of any commercial or financial relationships that could be construed as a potential conflict of interest.
